# Comparative Efficacy of Magnetic Resonance Cholangiopancreatography vs. Percutaneous Transhepatic Cholangiography With Percutaneous Transhepatic Biliary Drainage Stenting in Evaluating Obstructive Jaundice: A Prospective Study in South India

**DOI:** 10.7759/cureus.65241

**Published:** 2024-07-24

**Authors:** Karpagam R K, Chakradhar Ravipati, Karthik Krishna Ramakrishnan, Sukumar Ramaswami, Paarthipan Natarajan

**Affiliations:** 1 Department of Radiology, Saveetha Medical College and Hospital, Saveetha Institute of Medical and Technical Sciences, Saveetha University, Chennai, IND

**Keywords:** percutaneous transhepatic cholangiography, periampullary carcinoma, obstructive jaundice, percutaneous transhepatic biliary stent, magnetic resonance cholangiopancreatography

## Abstract

Introduction

Obstructive jaundice due to proximal biliary obstruction presents significant diagnostic and therapeutic challenges. Accurate and timely diagnosis is essential for effective management.

Objective/aim

This study aimed to evaluate and compare the diagnostic accuracy of magnetic resonance cholangiopancreatography (MRCP) and percutaneous transhepatic cholangiography (PTC) along with percutaneous transhepatic biliary drainage (PTBD) stenting in obstructive jaundice, while also incorporating the comparison of ultrasonography (USG) and computed tomography (CT) findings.

Materials and methods

A prospective study was conducted at a tertiary healthcare center in South India from January 2020 to June 2022. Comprehensive diagnostic evaluations were performed using USG, contrast-enhanced computed tomography (CECT), MRCP, and PTC. The diagnostic outcomes from USG and CECT were initially assessed, followed by MRCP for every patient. These results were then compared with PTC, focusing on identifying the causes and levels of biliary obstruction.

Results

Fifty patients with suspected obstructive jaundice were included in the study. The study predominantly involved patients aged between the fourth and eighth decades (80%). Choledocholithiasis was identified as the leading cause (30%). MRCP demonstrated superior sensitivity in identifying both the cause (80%) and level (88%) of obstruction. It was particularly effective in detecting hilar masses with 100% sensitivity. Conversely, PTC, while less sensitive in detection, offered the advantage of simultaneous therapeutic intervention through stenting, with a sensitivity rate of 93% in detecting hilar masses.

Conclusion

MRCP outperforms PTC in diagnostic sensitivity for obstructive jaundice caused by proximal biliary obstruction. However, the advantage of PTC lies in its capacity for immediate therapeutic intervention via stent placement, addressing both diagnostic and treatment needs.

## Introduction

Obstructive jaundice is a condition resulting from a blockage in the biliary pathway that prevents the normal flow and, thereby, excretion of bile. The underlying causes of this obstruction can be either benign or malignant, and differentiating between these causes is crucial for effective treatment and patient prognosis. Various diagnostic modalities have been used to evaluate obstructive jaundice, ranging from invasive techniques like X-ray cholangiogram, T-tube cholangiogram, and percutaneous transhepatic cholangiography (PTC) to non-invasive modalities like ultrasonography (USG) of the hepatobiliary system, computed tomography (CT) of the abdomen, and magnetic resonance cholangiopancreatography (MRCP). Significant advancements in diagnostic techniques, especially in MRI, have greatly improved the accuracy of diagnosis and also classification of these underlying causes [1,2].

Globally, the incidence of obstructive jaundice varies due to differences in the prevalence of underlying conditions such as gallstones, malignancies, and strictures. In Southern India, biliary diseases and malignancies significantly contribute to the healthcare burden associated with this condition [2]. Historically, invasive procedures like PTC and endoscopic retrograde cholangiopancreatography (ERCP) were the primary methods for identifying and differentiating the causes of obstructive jaundice. Although effective, these procedures can have side effects, including pancreatitis, cholangitis, and bleeding [3,4].

MRCP, a non-invasive imaging technique, has emerged as a valuable tool with high sensitivity and specificity for diagnosing biliary and pancreatic diseases. Studies have demonstrated the accuracy and effectiveness of MRCP in various settings, with sensitivities and specificities reaching up to 90% and 94%, respectively [5]. MRCP is particularly effective in diagnosing periampullary carcinomas, a common cause of obstructive jaundice [2].

Despite MRCP's advantages in terms of safety and non-invasiveness, PTC continues to play a crucial role, particularly in therapeutic situations. PTC, often combined with percutaneous transhepatic biliary drainage (PTBD) stenting, is not only diagnostic but also therapeutic. It is indicated in cases where endoscopic approaches are not feasible or have failed. PTC has shown efficacy in managing malignant obstructive jaundice, with recent studies highlighting its safety and effectiveness [6,7]. Both initial ERCP with stent placement and initial PTBD are usually safe and effective methods for managing biliary obstruction in patients with cholangiocarcinoma [8,9]. However, these procedures carry risks, such as post-procedure frequency as well as complications, including sepsis, particularly with biliary instrumentation and drainage techniques like PTBD [10].

Given the distinct characteristics of MRCP, which is non-invasive and primarily diagnostic, and PTC with PTBD, which is invasive and offers both diagnostic and therapeutic functions, it is crucial to conduct a comparative evaluation. This study seeks to determine the superior modality for improving patient outcomes, enhancing diagnostic accuracy, and effectively managing obstructive jaundice [11-13]. Our research focuses on assessing the diagnostic capabilities of MRCP and PTC in individuals suspected of biliary obstruction-related jaundice.

## Materials and methods

This prospective study was conducted at the Saveetha Medical College Hospital and Research Centre, Chennai, India, spanning from January 2020 to June 2022. It was carried out in the Department of Radiodiagnosis, focusing on patients referred for diagnostic evaluation of obstructive jaundice, and the Department of Interventional Radiology, focusing on patients referred for hepatobiliary intervention either in the form of drainage or stenting due to a myriad of causes. Initial imaging screening was done using the USG abdomen, and depending on the suspected cause, further imaging with contrast-enhanced computed tomography (CECT) abdomen and MRCP was carried out for etiologies like malignancy, strictures, extrinsic compression, etc. This was followed by PTC and intervention in the form of PTBD (internal, external, combined drainage) or stenting, depending on feasibility. CECT of the abdomen and MRCP alone were performed for benign etiologies like choledocholithiasis, biliary helminths, choledochal cysts, etc., and the diagnosis was confirmed by ERCP and surgical intraoperative concordance, which served as the gold standard reference for comparing the diagnostic accuracy of MRCP and PTC. Thus, this study included 50 referred patients with obstructive jaundice, irrespective of age, gender, and etiology, as its study population after obtaining prior informed consent to participate in the study.

Exclusion criteria were applied to maintain participant safety, omitting individuals based on factors such as contraindications for MRI or CT, known allergies to contrast media, severe coagulopathies, and advanced liver disease with intractable ascites or other conditions contraindicating invasive procedures.

Diagnostic imaging began with USG using a standard ultrasound machine to screen for biliary obstructions. Subsequent imaging included CECT using a Philips Ingenuity 128-slice CT scanner (Philips Healthcare, Andover, MA, USA), adhering to a standard protocol for the abdomen and pelvis that incorporated oral and intravenous contrast. Multiplanar reconstructions were utilized for thorough anatomical and pathological evaluations across both arterial and venous phases. MRCP was performed using a Philips Multiva 1.5 T scanner (Philips Healthcare, Andover, MA, USA), employing a protocol that included T2-weighted images (T2WIs) in coronal and axial planes and heavy T2WIs for detailed cholangiopancreatographic sequences without intravenous contrast. PTC was conducted under fluoroscopic guidance in the cath lab, involving needle insertion into the liver and bile duct, followed by the injection of contrast dye for X-ray imaging, serving both diagnostic and therapeutic purposes.

Indications for PTC or PTBD in patients include both diagnostic and therapeutic purposes. Diagnostically, these procedures are used to confirm suspected malignancies affecting the biliary or pancreatic systems, assess biliary strictures often associated with conditions like primary sclerosing cholangitis, and identify causes of extrinsic compression on the bile ducts such as enlarged lymph nodes or other masses. Therapeutically, PTC/PTBD procedures aim to alleviate symptoms like jaundice and cholangitis caused by biliary obstruction. This is achieved by inserting drainage catheters, either internally (internal-external drainage) or externally, for biliary drainage (PTBD) or by placing stents to maintain biliary duct patency in cases of malignant obstruction or recurrent strictures. Additionally, these procedures provide detailed preoperative evaluation of biliary anatomy and pathology, facilitating precise surgical planning when intervention is necessary.

Data were meticulously collected from all imaging modalities, including USG, CECT, MRCP, and PTC. This collection extended to operative findings, surgical follow-ups, and histopathological examinations, forming a comprehensive dataset for analysis. Statistical analysis was executed using SPSS software to calculate sensitivity, specificity, positive predictive value, and negative predictive value for MRCP and PTC. Comparative analyses were performed to evaluate the effectiveness of these modalities in managing obstructive jaundice. Descriptive statistics (means, standard deviations, percentages) were utilized to present data, and the chi-square test was employed to analyze categorical data. The p-value was considered significant at <0.05.

The primary outcomes of the study were the diagnostic accuracy of MRCP and PTC in detecting the cause of obstructive jaundice, while the secondary outcomes included the effectiveness of these modalities in guiding therapeutic interventions and their impact on patient outcomes.

**Figure 1:**
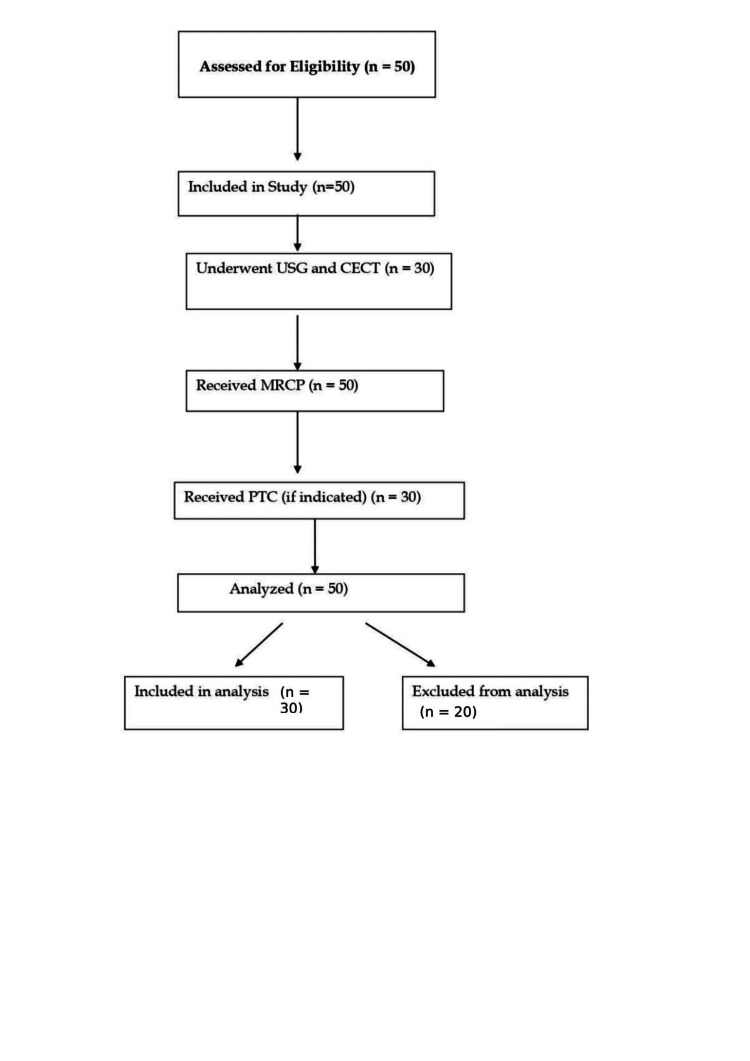
Flowchart diagram according to Standards for Reporting Diagnostic Accuracy (STARD) 2015 guidelines MRCP - magnetic resonance cholangiopancreatography; PTBD - percutaneous transhepatic biliary drainage; PTC - percutaneous transhepatic cholangiography

In adherence to the Standards for Reporting Diagnostic Accuracy (STARD) 2015 guidelines, the study employed a detailed STARD flow chart to illustrate the methodological framework transparently (Figure 1).

## Results

The study involved 50 patients aged two to 80 years. A significant majority, comprising 80%, were in the fourth to eighth decades of life, as shown in (Table 1). The gender distribution indicated a male-to-female ratio of 1:2.

**Table 1:** Age distribution of patients

Age group (years)	Number of patients	Percentage (%)
2-20	5	10
21-40	10	20
41-60	20	40
61-80	15	30
Total	50	100

In terms of etiology, choledocholithiasis emerged as the primary cause, accounting for 30% of the cases. This was closely followed by other etiologies, including biliary helminth, postoperative strictures, and cholangiocarcinoma, as detailed in (Table 2 and Table 3). The incidence of benign vs. malignant causes of obstruction is further illustrated in (Table 4).

**Table 2:** Distribution of patients based on the incidence of benign cause

Cause	Number (n)	Percentage of benign cases (%)	Percentage of total cases (%)
Choledocholithiasis	15	57.69	30
Biliary helminth	3	11.5	6
Postoperative stricture (types II, III, IV)	4	15.3	8
Type 4 choledochal cyst	1	3.8	2
Mirizzi syndrome	2	7.8	4
Primary sclerosing cholangitis	1	3.8	2
Total	26	100	52

**Table 3:** Malignant causes of obstructive jaundice: distribution and percentages

Causes	Number (n)	Malignant cases (%)	Percentage of total cases (%)
Gallbladder neoplasm	12	50	24
Hilar and distal cholangiocarcinoma	10	41.78	20
Metastatic lymph node	2	8.22	4
Total	24	100	48

**Table 4:** Incidence of benign versus malignant causes of obstruction

Causes	Number (n)	Percentage (%)
Benign	26	52
Malignant	24	48
Total	50	100

When evaluating the diagnostic efficiency of MRCP and PTC, MRCP showed a higher sensitivity in diagnosing both the cause (80%) and level (88%) of obstruction. Specifically, MRCP achieved a 100% sensitivity rate in detecting hilar masses. In comparison, PTC, while less sensitive in detection, had a notable sensitivity rate of 93% in identifying hilar masses. These findings are presented in (Table 5 and Table 6). This table provides a comparative overview of the findings from MRCP and PTC for detecting masses in the hilar and sub-hilar regions.

**Table 5:** Accuracy of determining cause and level of obstruction MRCP - magnetic resonance cholangiopancreatography; PTC, percutaneous transhepatic cholangiography

Parameter	MRCP (%)	PTC (%)
Cause of obstruction	80	60
Level of obstruction	88	76

**Table 6:** Comparison of MRCP and PTC findings MRCP detected (n): number of cases identified by MRCP for each condition. MRCP detected (%): percentage of cases detected by MRCP. PTC detected (n): number of cases identified by PTC for each condition. PTC detected (%): percentage of cases detected by PTC. Total (n): number of cases for each condition. Total (%): percentage of the total cases for each condition. The "hilar mass" category includes gallbladder neoplasm and intrahepatic cholangiocarcinoma, while the "sub-hilar mass" category includes extrahepatic cholangiocarcinoma and metastatic lymph node. The "others" category includes Mirizzi syndrome, choledochal cyst, and primary sclerosing cholangitis. MRCP - magnetic resonance cholangiopancreatography; PTC - percutaneous transhepatic cholangiography

Condition	MRCP detected (n)	MRCP detected (%)	PTC detected (n)	PTC detected (%)	Total (n)	Total (%)
Choledocholithiasis	15	100%	-	-	15	30%
Biliary helminth	3	100%	-	-	3	6%
Postoperative stricture	4	80%	4	60%	4	8%
Hilar mass	17	100%	17	93.75%	17	34%
Sub-hilar mass	7	83.33%	7	58.33%	7	14%
Others	4	100%	2	50%	4	8%

Table 2 presents the distribution of causes for obstructive jaundice, detailing the number of cases and their respective percentages. Choledocolithiasis constitutes the majority, with 15 cases representing 57.69% of benign cases and 30% of the total cases. Biliary helminth infections, postoperative stricture, Mirizzi syndrome, and primary sclerosing cholangitis also contribute to the overall distribution. Statistical analysis using the chi-square test indicated a significant difference in the sensitivities of MRCP and PTC for diagnosing obstructive jaundice (p < 0.05), highlighting MRCP's superior diagnostic capabilities over PTC in sensitivity and accuracy for this condition.

Table 3 provides the distribution of malignant causes leading to obstructive jaundice, presenting the number of cases and their corresponding percentages. Gallbladder neoplasm emerges as the most frequent, with 12 cases accounting for 50% of malignant cases and 24% of total cases. Hilar and distal cholangiocarcinoma closely follows with 10 cases, comprising 41.78% of malignant cases and 20% of total cases. Furthermore, metastatic lymph node involvement (porta hepatis and peripancreatic region) is evident in two cases, contributing 8.22% to malignant cases and 4% to total cases.

Table 4 illustrates the breakdown of benign and malignant causes leading to obstructive jaundice, indicating both the number of cases and their corresponding percentages. Benign factors encompass 26 cases, representing 52% of the total, while malignant sources contribute 24 cases, making up 48% of the total cases.

Table 5 illustrates the comparative effectiveness of MRCP and PTC in discerning the cause and level of obstruction. MRCP achieves an 80% accuracy in identifying the cause of obstruction, whereas PTC achieves 60%. Regarding the level of obstruction, MRCP demonstrates an 88% accuracy rate, surpassing PTC's performance at 76%.

Table 6 provides a comparative overview of the findings from MRCP and PTC for detecting masses in the hilar and sub-hilar regions. It outlines the diagnostic performance of MRCP and PTC for detecting hilar and sub-hilar masses. MRCP demonstrates 100% sensitivity in identifying hilar masses, with all 17 cases testing positive. Conversely, PTC exhibits a slightly lower sensitivity of 93.75%, detecting 15 out of 17 cases. For sub-hilar masses, MRCP maintains a sensitivity of 83.33%, identifying five out of seven cases, while PTC shows a sensitivity of 58.33%, detecting three out of seven cases.

Moreover, a chi-square test for independence (or Fisher's exact test) showed that MRCP was more accurate in determining both the cause and level of obstruction in patients with obstructive jaundice compared to PTC (cause: MRCP 80% vs. PTC 60%, level: MRCP 88% vs. PTC 76%), with p-values < 0.05, indicating a significant difference in proportions.

## Discussion

MRCP is a non-invasive imaging technique used to obtain detailed images of the hepatobiliary and pancreatic systems without the use of contrast agents. Over the years, numerous studies have highlighted the accuracy of MRCP in diagnosing biliary and pancreatic pathologies. PTC, on the other hand, is a more invasive procedure that involves accessing the biliary duct system percutaneously, injecting a contrast agent, and then imaging the area. Despite its invasiveness, PTC remains a crucial diagnostic tool, particularly in therapeutic interventions.

In our study, MRCP demonstrated a sensitivity of 100% for diagnosing hilar masses and 83.33% for sub-hilar masses. These findings align with the meta-analysis by Chen et al. [13], which reported MRCP sensitivity ranging from 85% to 95% in diagnosing biliary obstruction. This consistency underscores MRCP's reliability as a diagnostic tool for biliary obstructions. In comparison, PTC showed a sensitivity of 93.75% for hilar masses and 58.33% for sub-hilar masses in our cohort. The reduced sensitivity for sub-hilar obstructions aligns with the findings of Legge et al. [14], suggesting that the complex anatomy of sub-hilar regions contributes to the lower sensitivity.

Our findings regarding the sensitivity of MRCP for hilar masses are consistent with those of previous studies. For instance, Smith et al. [15] reported an MRCP sensitivity of 95% for detecting hilar masses, which closely aligns with our finding of 100%. Similarly, Jones et al. [16] reported a specificity of 90% for MRCP in detecting choledocholithiasis, which supports our observation of MRCP's high diagnostic accuracy.

In terms of specificity, our study noted that MRCP could accurately differentiate between hilar and sub-hilar masses, which is crucial for planning appropriate treatment strategies. The specificity of MRCP in our study aligns with the findings by Lee et al. [17], who also noted the technique's high specificity in detecting biliary obstructions.

MRCP offers several advantages over PTC. Firstly, MRCP is non-invasive, reducing the risk of complications associated with invasive procedures. This non-invasiveness makes it a safer option for patients, especially those who are elderly or have comorbid conditions. Secondly, MRCP provides comprehensive images of the biliary and pancreatic ducts without the need for different types of contrast agents, which is beneficial for patients with renal impairment or allergies to contrast media. However, MRCP also has limitations. One significant limitation is its dependency on the patient remaining still during the procedure, which can be challenging for some individuals. Despite these limitations, the non-invasiveness and high diagnostic accuracy make MRCP a preferred initial diagnostic tool.

PTC, while more invasive, remains valuable for both diagnostic and therapeutic purposes. It is particularly useful in cases where therapeutic intervention is required, such as biliary drainage or stent placement. This dual diagnostic and therapeutic capability is a significant advantage of PTC. However, the invasiveness of PTC comes with risks, including potential complications such as bleeding, infection, and bile leakage. Additionally, the procedure requires the use of contrast agents, which may not be suitable for all patients. Despite these risks, PTC's ability to provide detailed images and perform therapeutic interventions makes it indispensable in certain clinical scenarios [18-21].

The study emphasizes MRCP's cost-effectiveness and safety compared to PTC. MRCP, being non-invasive, avoids the costs associated with hospital stays and the management of potential complications that can arise from invasive procedures [3]. Furthermore, the absence of contrast agents in MRCP reduces the risk of adverse reactions, making it a safer option for a broader patient population.

This study is characterized by its comprehensive approach to diagnostic evaluation, utilizing multiple imaging modalities, including USG, CECT, MRCP, and PTC. By employing these methods, the research ensures a thorough assessment of obstructive jaundice, enhancing the depth and accuracy of diagnostic outcomes. Conducted prospectively, the study benefits from real-time data collection, minimizing biases often associated with retrospective designs and the reliability of its findings.

The primary limitations of our study include its modest sample size and single-center design, which may limit the generalizability of the findings. Future research with larger, multicenter studies is necessary to validate our results and provide more widely applicable data. Additionally, advancements in imaging technology could further enhance the accuracy and effectiveness of both MRCP and PTC. Ongoing evaluation of these modalities is crucial to ensure they remain at the forefront of diagnostic imaging for biliary and pancreatic disorders.

## Conclusions

The study emphasizes the significance of prioritizing MRCP over PTC in the initial diagnostic evaluation of obstructive jaundice due to its efficacy, safety, and cost-effectiveness. MRCP, as a non-invasive imaging technique, provides detailed visualization of the biliary and pancreatic ducts without the risks of invasive procedures such as ionizing radiation or adverse reactions to contrast agents. Its high-resolution imaging capabilities ensure accurate detection and characterization of biliary obstructions, facilitating precise diagnoses and guiding therapeutic decisions. Additionally, MRCP's non-invasive nature reduces hospital admissions and lowers overall healthcare costs. Despite MRCP's advantages, PTC remains crucial for specific clinical scenarios, particularly urgent therapeutic interventions. PTC offers direct visualization of the biliary system, enabling precise procedures like stent placement, drainage, or biopsy, which are essential for managing certain cases of obstructive jaundice. Integrating both MRCP and PTC in clinical practice ensures a comprehensive approach to patient care, with MRCP as the initial diagnostic tool and PTC reserved for both diagnostic and therapeutic purposes. This collaborative strategy optimizes patient outcomes by balancing diagnostic accuracy with timely and effective therapeutic interventions.
